# Game over too soon: early specialization and short careers in esports

**DOI:** 10.3389/fpsyg.2025.1585599

**Published:** 2025-05-12

**Authors:** Jimoon Kang, Seongcheol Kim

**Affiliations:** College of Media and Communication, Korea University, Seoul, Republic of Korea

**Keywords:** esports, early specialization, fragmented governance, career longevity, burnout, doping, athlete welfare

## Abstract

**Introduction:**

Esports has become a global phenomenon, offering considerable professional opportunities, enhanced cognitive abilities, and strong social capital for young competitors. However, these benefits are often counterbalanced by significant challenges, including burnout, exploitation, and inconsistent governance, particularly for its predominantly young talent.

**Methods:**

In this convergent mixed-methods study, we examine the factors influencing career longevity in esports by integrating a longitudinal analysis of 15,021 players’ records (1998–2023) and in-depth qualitative interviews conducted with ten key stakeholders in the Korean esports ecosystem.

**Results:**

Our quantitative findings reveal an exponential increase in tournaments, prize money, and active competitors yet also expose a concerning trend: newer birth cohorts, especially those born after 1998, exhibit markedly shortened careers—with median durations approaching just 2 years. Complementary qualitative insights elucidate how early specialization, exploitative contractual practices, intense training regimens, and fragmented governance contribute to burnout and rapid career turnover.

**Discussion:**

Together, these results underscore the urgent need for comprehensive policy reforms, including standardized contractual frameworks, holistic athlete support systems, and centralized regulatory oversight, to safeguard young talent and ensure the long-term sustainability of esports.

## 1 Introduction

At just 15 years old, many aspiring esports prodigies are already practicing upwards of 10 h a day, driven by the promise of fame and fortune—yet, many find themselves sidelined or burned out before they even turn 20. Over the past two decades, esports has undergone a meteoric transformation from small-scale gaming contests to a billion-dollar global phenomenon (Scholz, 2019; Kim and Kim, 2022). Along the way, it has amassed legions of dedicated spectators, built sophisticated infrastructure for training and coaching, and awarded tournament prize pools rivaling those in traditional professional sports (Ahn et al., 2020). Despite the striking “light” aspects of esports—such as enhanced cognitive skills, abundant career opportunities, and thriving online communities—this surging industry also grapples with a “dark” undercurrent. Young competitors, often minors, routinely log excessive practice hours, sign contracts with inadequate safeguards, and face volatile working conditions that can heighten mental stress and physical wear (Kari and Karhulahti, 2016; DiFrancisco-Donoghue et al., 2019; Poulus et al., 2024a; Poulus et al., 2024b).

Evidence of this dichotomy has emerged in the phenomenon of early specialization wherein adolescent players devote themselves exclusively to one game. On the one hand, this singular focus can rapidly sharpen reaction times, strategic thinking, and hand–eye coordination—arguably a triumph in terms of the potential benefits of gaming (Zhong et al., 2022). On the other hand, it can also intensify health risks and psychological strain. Such vulnerability is compounded by esports’ fragmented governance, where game publishers, regional leagues, and private organizers wield disparate authority. Consequently, players receive uneven protection in areas such as contractual fairness, anti-doping protocols, and mental health support (Holden et al., 2017; Salum et al., 2024; Poulus and Polman, 2022; Leis et al., 2024).

This fragmentation stems largely from the unique position of game publishers as both intellectual property owners and de facto regulatory authorities—a structure fundamentally different from traditional sports. While conventional sports separate equipment manufacturers from governing bodies, esports grants publishers unprecedented control over competitive environments, creating what Bulut (2020) terms “ludopolitics.” Publishers can unilaterally modify game mechanics, tournament structures, and competitive formats, introducing instability that affects player careers regardless of skill or dedication. These conditions parallel challenges documented in game development labor by O’Donnell (2014), and Legault and Weststar (2017), as well as in game-adjacent industries like streaming and content creation (Cote and Harris, 2021; Keogh, 2019), where technological change and aspirational labor exist alongside minimal regulatory protections. The result is an ecosystem where precarity and opportunity are inextricably linked.

As a result, while some participants thrive in well-financed organizations that emphasize player well-being and balanced schedules, many others languish in exploitative or poorly regulated environments. Thus, esports epitomizes a broader debate within media psychology: do digital games chiefly offer enrichment—via improved cognition, social capital, and recreational enjoyment—or do they engender hazards such as addictive behaviors, aggressive interactions, and compromised integrity (Macey and Hamari, 2019; Kowert, 2020)?

Despite increasing scholarly and public interest, much of the existing esports research remains limited to individual game titles or short-term outcomes (Rudolf et al., 2020; Overå et al., 2024). A more holistic perspective is needed to capture how these “light” and “dark” dynamics shape longer-term career paths—particularly for the youngest competitors—amid rapidly changing industry conditions. In particular, there is a pressing need to understand how the “light” drivers of success might coexist with, or be undermined by, the “dark” realities of contractual instability, doping concerns, and minimal athlete protections. To address this gap, the present study employs a convergent mixed-methods approach, combining a longitudinal dataset of over 15,000 esports players with in-depth qualitative interviews conducted in the Korean esports ecosystem. Our findings illuminate not just the scale of early burnout, where short careers are increasingly the norm, but also the structural weaknesses that perpetuate high turnover. In so doing, we offer policy and governance recommendations aimed at cultivating a safer, more sustainable playing field that preserves the best of what digital gaming has to offer while mitigating its most harmful outcomes.

## 2 Literature review

### 2.1 Esports growth and governance

Esports has experienced an extraordinary transformation since its modest beginnings in the late 1990s. What once started as localized, grassroots tournaments has rapidly evolved into a global industry that attracts millions of viewers and billions of dollars in investment (Scholz, 2019; Taylor, 2012). Advances in live-streaming technology, enhanced audience engagement, and lucrative sponsorship opportunities have driven exponential increases in prize pools and professional player participation (Macey and Hamari, 2019; Reitman et al., 2020). Recent market projections estimate that esports revenues will approach nearly USD 4.8 billion in 2025, highlighting the remarkable commercial impact and widespread appeal of competitive gaming (Statista, 2024; Ahn et al., 2020).

However, the rapid professionalization has exposed a key structural tension: while game publishers, private organizers, and regional associations have each contributed to the industry’s expansion, they also operate with disparate priorities and rules (Hallmann and Giel, 2018; Scholz, 2019). Unlike traditional sports leagues, which often follow unified regulatory frameworks, esports relies on a fragmented governance model wherein intellectual property rights rest primarily with game developers. This decentralized environment confers certain benefits—such as creative freedom, swift adaptation to emerging technologies, and the rapid growth of supportive online communities—but also poses significant risks in terms of player welfare (Holden et al., 2017).

Further complicating this regulatory patchwork, independent organizers such as ESL and DreamHack have established their own sets of rules or collaborated voluntarily with oversight bodies like the Esports Integrity Commission (ESIC) (ESIC, 2020). This pluralistic system has prompted certain regions to attempt a more consolidated approach. For example, in South Korea, national esports federations work in tandem with government agencies to manage player licensing, event sanctioning, and youth protection measures (Heere, 2018). However, these coordinated efforts often encounter challenges when they overlap with the decentralized, publisher-driven structures that dominate international competitions.

A prime negative consequence of this fragmented landscape is the absence of universal safeguarding measures. Many semi-professional or academy-level tournaments are not overseen by established bodies like ESIC, meaning that standards for player contracts, working conditions, and youth protections can vary dramatically (ESIC, 2020; Macey and Hamari, 2019). Young competitors—often minors—are particularly vulnerable to the effects of inadequate rest, erratic practice schedules, and minimal health resources. Conversely, smaller independent events can spur local innovation and community building, highlighting how governance fragmentation can simultaneously foster a “light” pathway for new talent while exposing others to “dark,” under-regulated environments. The challenge, therefore, is finding a regulatory balance that preserves these creative opportunities while mitigating exploitation and uneven oversight.

### 2.2 Early specialization

Early specialization, defined as the intensive focus on one sport or activity from a young age, has become a hallmark of competitive esports (Kari and Karhulahti, 2016). In traditional sports, early specialization is often a deliberate strategy aimed at harnessing the plasticity of youth to develop elite skills. In esports, however, the pressure to specialize early is compounded by unique industry dynamics, such as the rapid pace of technological change and the high cognitive demands of competitive gaming (Zhong et al., 2022).

On the positive side, early specialization in esports can accelerate skill acquisition. Young players, often beginning in their early teens, benefit from the developmental advantages of enhanced reaction times and cognitive flexibility, which are critical in fast-paced, high-stakes gaming environments (DiFrancisco-Donoghue et al., 2019). Intensive, focused training regimes can result in the rapid honing of strategic thinking and technical skills, providing an early pathway into professional circuits. This phenomenon is supported by evidence from training studies in both esports and traditional sports, which suggest that sustained, deliberate practice is key to achieving high-level performance (Erickson and Côté, 2016).

However, the benefits of early specialization entail considerable risks. One primary concern is the heightened risk of burnout and overuse injuries. In esports, the intense practice schedules—often exceeding 10–14 h per day—can lead to chronic physical issues, such as musculoskeletal strain in the wrists, fingers, and lower back (Kari and Karhulahti, 2016). Unlike traditional athletes, esports competitors may have limited access to structured physical conditioning and rest protocols, which increases their vulnerability to injuries that can prematurely end their careers.

Moreover, the psychological toll of early specialization should not be underestimated. The high-pressure environment of competitive gaming, coupled with constant public scrutiny via live streaming and social media, contributes to significant mental health challenges. Research has demonstrated various patterns of burnout among esports players (Poulus et al., 2024a), with resilience and coping strategies playing crucial roles in sustainability (Poulus and Polman, 2022; Leis et al., 2024). Young players often face performance anxiety, social isolation, and a pervasive fear of failure, which can precipitate early burnout (DiFrancisco-Donoghue et al., 2019; Leis et al., 2022). Recent network analysis approaches have revealed how burnout components interact with resilience and coping mechanisms, suggesting that intervention strategies need to target multiple psychological dimensions simultaneously (Poulus et al., 2024b).

The implications of early specialization extend beyond individual health, impacting the broader sustainability of esports as an industry. As studies have demonstrated, the careers of esports players, particularly those who begin competing at a young age, tend to be markedly shorter than those of traditional sports players (Rudolf et al., 2020). This trend suggests that while early specialization may offer a competitive edge in the short term, it also creates a talent pipeline characterized by rapid turnover, potentially undermining the long-term stability of the industry.

### 2.3 Doping and integrity issues

Doping and integrity challenges represent a multifaceted issue in esports that is both similar to and divergent from that found in traditional sports. In conventional athletics, doping typically involves the misuse of anabolic steroids or hormones; by contrast, performance-enhancing practices in esports more commonly involve stimulants (e.g., Adderall, Ritalin) designed to sharpen concentration, reaction times, and cognitive endurance (Fashina, 2021; Salum et al., 2024). While these substances offer a competitive edge in an environment where even millisecond differences can be decisive, their use raises serious ethical and health concerns, particularly among younger players who may lack full awareness of the risks involved.

The governance landscape further complicates the issue. Although some major esports leagues have begun to introduce anti-doping protocols in collaboration with bodies like ESIC (ESIC, 2020), the broader esports ecosystem remains highly fragmented. Many tournaments, especially those at the regional or amateur levels, do not enforce comprehensive testing measures, leaving significant gaps in regulation and accountability (Holden et al., 2017). This uneven enforcement creates pockets within the industry where the use of performance-enhancing substances can go unchecked, thereby undermining the competitive integrity of the sport.

Beyond the misuse of stimulants, integrity challenges in esports also include issues such as match-fixing, cheating via software exploits, and the manipulation of player identities. The absence of a centralized governing body akin to the World Anti-Doping Agency (WADA) in traditional sports exacerbates these issues, resulting in a lack of uniform standards and penalties across different competitions (Scholz, 2019). In this environment, regulatory measures are often reactive rather than proactive, and inconsistent enforcement further erodes trust among stakeholders.

Hence, while doping exemplifies the “dark” side of performance pressures, a coordinated, transparent regulatory system could help preserve the “light” side of fair competition and skillful play. Without coordinated and industry-wide efforts to implement robust anti-doping policies and enforce integrity standards, the credibility of esports as a professional sport may continue to be undermined.

### 2.4 Career longevity

Although the question of career longevity in esports has garnered increasing attention as the industry matures, it remains one of the least understood aspects of competitive gaming. Early evidence suggests that in contrast to traditional sports, where structured athlete development can support prolonged careers (Varghese et al., 2022; Erickson and Côté, 2016), esports players often experience notably short career spans (Rudolf et al., 2020; Overå et al., 2024). Many professional competitors enter the scene in their late teens or early twenties only to exit after just two or three competitive seasons—a trend that raises crucial questions about sustainability and player welfare.

Several factors contribute to these shortened career trajectories. The intensive demands of early specialization, including grueling practice schedules and high cognitive stress, can accelerate burnout and lead to premature career termination (Kari and Karhulahti, 2016; DiFrancisco-Donoghue et al., 2019). Additionally, the fragmented governance structure in esports may exacerbate career volatility, as inconsistent contractual standards and minimal regulatory oversight can leave young players particularly vulnerable to abrupt dismissals or exploitative practices (Holden et al., 2017; Macey and Hamari, 2019). Moreover, the rapid pace of competitive performance and the significant impact of marginal performance declines often mean that even a slight dip in ability can result in contract termination or relegation, further shortening professional careers (Rudolf et al., 2020).

Recent research has begun to explore interventions that might extend career longevity. Studies on physical exercise and performance in esports have demonstrated potential benefits of structured physical training regimens (McNulty et al., 2023), while investigations into self-determination toward exercise suggest that intrinsic motivation for physical activity may contribute to sustainability (Nicholson et al., 2024a). Preliminary evidence also indicates that targeted exercise interventions may positively affect cognitive function and physiological markers like heart rate variability in elite players (Nicholson et al., 2024b). These findings suggest that holistic approaches to player development, integrating both physical and cognitive dimensions, may help counteract some factors contributing to short careers.

Despite these concerns, existing research on esports career longevity is limited. Many studies have focused on specific game titles or small samples, which may not capture the full range of experiences across the diverse esports landscape (Hollist, 2015; Kari and Karhulahti, 2016). Furthermore, while traditional sports have benefited from decades of longitudinal data and athlete development research, esports research remains nascent, often relying on cross-sectional analyses or datasets that predominantly track high-profile tournaments (Lee and Schoenstedt, 2011; Scholz, 2019). This gap is particularly significant given the exponential growth in esports prize pools and sponsorships, which has raised both the stakes and the risks associated with early entry into professional competition.

These research limitations are further complicated by significant regional variations in esports ecosystems. While our current understanding of career longevity is constrained by methodological challenges, emerging comparative studies suggest that institutional and cultural contexts play crucial roles in shaping player experiences. Research on collegiate esports in Western contexts (Harris et al., 2022; Wilson et al., 2024; Cote et al., 2023) reveals patterns of exploitation and opportunity similar to those in professional circuits, albeit within different regulatory frameworks. These studies highlight how institutional structures can either mitigate or exacerbate the precarity inherent in early specialization. Similarly, Can and Foxman (2021) have documented how Turkish players create ad-hoc spaces to navigate contextual challenges, illustrating how regional adaptations emerge in response to governance gaps. Hence, what might appear as a universal “dark” pattern of rapid burnout can be partially mitigated by robust systems of player support—offering a “light” counterbalance that varies from region to region.

### 2.5 Research questions

Taken together, the previous sections illustrate how esports embodies two parallel and sometimes competing dynamics. On the “light” side, its rapid growth, accessible digital infrastructure, and evolving professional support systems can accelerate skill acquisition and cognitive development and offer social opportunities for aspiring players (Zhong et al., 2022; Varghese et al., 2022). On the “dark” side, however, fragmented governance, unregulated doping practices, and the pressure cooker of early specialization often produce adverse physical and psychological outcomes (DiFrancisco-Donoghue et al., 2019; Fashina, 2021). While some organizations have begun adapting policies from traditional sports medicine—such as employing performance analysts and sports psychologists—there is no universal framework mandating consistent player support or standardized contractual protections (Holden et al., 2017; Scholz, 2019).

In this dual context, any examination of esports must consider not only its commercial and developmental promise but also its structural vulnerabilities. Early specialization magnifies both poles of this continuum: while it can supercharge a player’s skill trajectory, it may also heighten their susceptibility to overuse injuries, fatigue, and exploitative practices. Similarly, doping and integrity issues reflect a governance gap that, left unchecked, can erode the trust and wellbeing of players across multiple tiers of competition. Together, these factors contribute to the characteristic short and volatile career arcs that define the esports landscape, setting the stage for mixed outcomes that defy simple categorization as either purely beneficial or detrimental.

Based on these intersecting themes, the present study pursues the following research questions:

- RQ1: What trends in player demographics, tournament frequency, and prize money characterize the rapid professionalization of esports from 1998 to 2023, and what are the implications of these trends for the industry’s growth?
- RQ2: How do career trajectories differ across birth cohorts in esports, and what role do factors such as early specialization and intensive training demands play in the accelerated burnout observed among younger competitors?
- RQ3: How do key stakeholders perceive the impact of fragmented governance, exploitative contractual practices, and inconsistent support systems on player welfare, and what policy reforms do they propose to enhance career sustainability in esports?

By addressing these questions, we aim to illuminate the coexistence of positive and negative dimensions within the esports environment, thereby offering a more nuanced perspective that can inform future policy reforms and sustainability efforts.

## 3 Methods

This study adopts a convergent mixed-methods design (Creswell and Plano Clark, 2017), integrating a large-scale quantitative investigation with qualitative interviews to explore the interplay between early specialization, governance conditions, and career trajectories in esports. The quantitative study analyzes a longitudinal dataset of esports players, while the qualitative study comprises in-depth interviews with key stakeholders in the Korean esports ecosystem. The purpose of this convergence is to generate a multifaceted understanding of how “light”-side developments (e.g., skill acquisition, community support) and “dark”-side challenges (e.g., burnout, doping) coalesce in shaping professional esports careers.

### 3.1 Quantitative study

Our quantitative analysis systematically explores the career trajectories of esports players over a 25-year period (1998–2023).

#### 3.1.1 Data sources and sample selection

This study’s quantitative component draws on publicly available tournament records from the *Esports Earnings*, covering 1998 to 2023. Data was accessed programmatically through the Esports Earnings API, adhering strictly to the platform’s terms of service. We utilized the LookupHighestEarningPlayers, LookupPlayerTournaments, and LookupRecentTournaments endpoints with appropriate rate limiting. *Liquipedia* was used exclusively for cross-reference validation of player information and career timelines.

Initial extraction yielded 145,825 records documenting participation in major esports titles. Data cleaning and cross-referencing with team announcements, official esports news outlets, and player social media accounts in Supplementary materials ensured the removal of duplicate entries and the resolution of conflicting birth years. Ultimately, the final analytic sample comprised 15,021 unique players with verified age information in Table 1, enabling robust age-based and career-length analyses.

**Table 1:** Data categories and access methods.

Category	API endpoint	Variables extracted	Cleaning procedure
Player demographics	LookupHighestEarningPlayers	PlayerId, NameFirst, NameLast, CurrentHandle, CountryCode, Birth Year	Cross-reference with Liquipedia for validation
Tournament participation	LookupPlayerTournaments	TournamentName, EndDate, GameId, Prize, TeamPlayers	Remove duplicates, standardize tournament naming
Prize money	LookupPlayerTournaments	Prize, ExchangeRate, CurrencyCode	Convert to USD using provided exchange rates
Player career information	LookupPlayerById	WorldRanking, CountryRanking, TotalUSDPrize, TotalTournaments	Cross-reference with Liquipedia for validation

#### 3.1.2 Measures

We employed several key measures to assess the impact of early specialization on career longevity in esports. First, we reviewed player birth year and tournament participation data to calculate the age at which each individual first entered competitive play, which allowed us to identify generational trends in entry and peak performance. Each player’s age at the time of competition was calculated by subtracting the birth year from the year of the tournament. We then categorized players into birth cohorts (e.g., 1986–1990, 1990–1994, etc.) to track shifts in career length across different generations (Rudolf et al., 2020). We determined career duration by measuring the time span between a player’s first and last recorded tournament, with players who were active at the study’s cutoff date treated as right-censored data to ensure methodological rigor.

We assessed financial success in esports based on cumulative prize earnings, which, while not adjusted for inflation, provided a comparative indicator of competitive achievement across different eras. To further explore patterns of engagement, we analyzed tournament participation counts as a proxy for professional activity, which revealed variations in competitive intensity over time. These measures collectively enabled a robust examination of how early specialization intersects with career sustainability and financial success.

#### 3.1.3 Analytical procedures

We employed a comprehensive analytical framework to examine the relationship between early specialization, career duration, and competitive performance trends in esports. Our analysis was structured around three key methodological components—descriptive statistics, non-linear modeling, and survival analysis—each designed to capture distinct dimensions of career trajectories.

Descriptive statistics provided an initial overview of career lengths, earnings distributions, and tournament participation rates. Measures of central tendency (mean, median) and dispersion (standard deviation, interquartile range) were computed to summarize the characteristics of esports players across different birth cohorts. To visualize the shifting patterns in age distributions and career lengths over time, we generated histograms and box plots to visualize variations across birth cohorts and observe how age distributions and career durations shifted over time.

To analyze peak performance ages and skill sustainability, we applied generalized additive models (GAMs), which enabled us to identify non-linear relationships between player age and competitive success. Unlike traditional regression models, which impose strict linear or polynomial assumptions, GAMs utilize smoothing splines to detect nuanced trends in career trajectories. The dependent variables in these models included cumulative prize earnings and tournament participation rates, with player age serving as the independent variable. Penalized regression splines were implemented with a smoothing parameter selected via restricted maximum likelihood (REML) to prevent overfitting. The effective degrees of freedom (EDF) were reported to indicate model flexibility, with values typically ranging between 3 and 6, suggesting a moderate degree of non-linearity in the relationship between age and performance.

To systematically assess career attrition, we employed Kaplan–Meier survival analysis, which provided non-parametric estimates of the probability of an esports player remaining active over time. Given the right-censored nature of the dataset—where players still competing as of December 2023 had incomplete career trajectories—Kaplan–Meier estimation was particularly well-suited for modeling time-to-event data. Survival curves were stratified by birth cohort to evaluate generational shifts in career longevity. We performed a log-rank test with a significance threshold of 0.05 to assess statistical differences between groups and computed hazard ratios using Cox proportional hazards models to quantify the relative risk of career termination across different birth cohorts.

To ensure the reliability of the findings, we also performed sensitivity analyses at multiple landmark intervals (30, 60, 90, and 180 days post-initial participation). These analyses were designed to mitigate biases stemming from early career dropouts and assess whether career trajectories were significantly influenced by a player’s initial rate of competitive engagement.

We performed all analyses in Python, utilizing libraries including pandas for data manipulation, numpy for numerical computations, matplotlib and seaborn for visualization, pygam for GAM analysis, and lifelines for survival analysis.

### 3.2 Qualitative study

While quantitative analyses can provide an industry-wide perspective on esports careers, they do not fully capture the lived experiences and operational nuances that influence these trajectories. To complement the numerical data, we conducted a qualitative strand consisting of semi-structured interviews with key stakeholders in the Korean esports ecosystem. This approach aligns with convergent mixed-methods principles (Creswell and Plano Clark, 2017), enabling a deeper exploration of the subjective realities embedded in professional gaming culture.

#### 3.2.1 Participant recruitment and sampling

We employed a purposive sampling strategy to identify individuals who possessed direct, hands-on experience with esports governance, coaching, and player management in South Korea. Potential participants were initially contacted via professional networks, organizational websites, and referrals within esports circles. Priority was given to assembling a balanced set of informants, including current and former players, coaches at both the academy and professional levels, team managers, and journalists reporting on the industry. This diversity was considered essential for uncovering a wide range of viewpoints regarding youth training regimens, early specialization pressures, and overall governance challenges. The final sample consisted of 10 participants, whose background information is summarized in Table 2.

**Table 2:** Interview participant information.

#	Category	Description
1	Professional coach	Current head coach of a top-tier Korean esports team; previously served as a pro player in the same top-level circuit.
2	Academy coach	Currently coaching at an academy program for a Korean esports organization; formerly played professionally at the top level.
3	Academy coach	Currently coaching in an academy/development league under a top-tier Korean esports team; previously a professional coach for a top-level roster.
4	Former professional player	Competed as a professional esports player in a top-tier Korean league; no longer active as a player.
5	Analyst	Currently serving as an analyst for a top-tier Korean esports team, with responsibilities in strategy and performance review.
6	General manager	Holds the position of General Manager for a top-tier Korean esports organization, overseeing team operations and management.
7	Professional player	Actively playing in a top-tier Korean League of Legends league, recognized internationally for its highly competitive standards.
8	Professional player	Active competitor in the secondary-tier (Challengers) league under the top-tier Korean League of Legends system.
9	Esports journalist	A journalist specializing in esports coverage.
10	Former professional coach	Served as a head coach for a top-tier Korean esports team; currently not coaching a professional roster.

#### 3.2.2 Interview process and data collection

To complement the quantitative analysis and provide a deeper understanding of the factors influencing career trajectories in esports, we conducted semi-structured interviews with key stakeholders in the Korean esports ecosystem. The interview guide in Supplementary materials was developed based on the themes identified in existing literature on esports careers and governance with our preliminary findings from quantitative analysis to capture insights into governance structures, player development pathways, training regimens, and the broader implications of early specialization.

The interviews were conducted through a combination of in-person meetings and secure video conferencing, depending on participant availability. Each session lasted between 15 and 60 min and followed a semi-structured format to ensure consistency while allowing flexibility for follow-up questions on emergent themes. Participants were provided with an overview of the study’s objectives and assured of their anonymity to encourage candid responses.

The interview framework was designed to explore several core areas relevant to career sustainability in esports. The questions covered governance and contractual practices, focusing on how regulatory structures impact player welfare, career stability, and competitive integrity. Another key area of inquiry addressed early specialization, investigating how training regimens, physical and psychological demands, and organizational structures influence player longevity. Discussions also covered integrity issues such as anti-doping measures, ethical concerns, and the enforcement of competitive fairness standards.

All interviews were recorded with participant consent and subsequently transcribed verbatim for analysis. We performed a thematic analysis to identify recurring patterns in stakeholder perspectives, with codes systematically grouped into broader categories to facilitate a structured interpretation of the data. Particular attention was given to areas where qualitative insights complemented or diverged from the quantitative findings. To ensure analytical rigor, intercoder reliability was assessed and emergent themes were iteratively refined through a comparative review process.

#### 3.2.3 Data analysis

Transcribed interviews underwent inductive thematic analysis following Braun and Clarke’s (2006) six-step approach: (1) familiarization with the data, (2) generating initial codes, (3) searching for themes, (4) reviewing themes, (5) defining and naming themes, and (6) producing the report. This approach aligns with our constructivist epistemological stance, recognizing that meaning emerges through the interaction between researchers and participants’ experiences. Initial open coding identified repeated phrases or ideas reflecting both negative (e.g., exploitative contracts, overtraining) and positive (e.g., skill mastery, social bonding) facets of esports. These codes were then sorted into broader thematic categories—such as “organizational support structures” and “mental health challenges”—and further refined based on frequency and conceptual overlap (Macey and Hamari, 2019).

Analytic memos were maintained to document reflexive observations and to cross-check emerging themes against the quantitative findings. This iterative approach enabled the researchers to construct a composite view of the stakeholder experience, revealing how issues like doping, fragmented governance, and the allure of rapid success shape the industry’s dual character. Finally, themes were compared across participant roles (player, coach, manager) to detect any role-specific perspectives on early specialization and potential reforms.

The integration of these qualitative insights with those gained from the longitudinal database analysis offers a richer, more nuanced understanding of esports’ evolving landscape. While the numeric data highlight large-scale trends in career duration and attrition, the qualitative interviews demonstrate how governance gaps, intense training regimens, and variably applied anti-doping measures may converge to either reinforce or undercut the “light”-side potential of a burgeoning digital sport (Tables 3, 4).

**Table 3:** Median survival times by birth cohort and landmark time.

Birth	Landmark time (Days)				
Cohort	30	60	90	180	365
−1986	3.77	3.77	3.74	3.7	3.68
1986–1990	4.42	4.38	4.3	4.3	4.47
1990–1994	4.54	4.59	4.59	4.66	4.65
1994–1998	4.06	4.01	4.01	3.95	3.81
1998–2002	3.27	3.23	3.17	3.05	2.81
2002–	2.43	2.37	2.32	2.21	1.93

**Table 4:** Median survival times by birth cohort and landmark time.

Cohort 1	Cohort 2	*p*_value	test_statistic
−1986	1986–1990	7.44E-01	0.106328
−1986	1990–1994	2.11E-01	1.563835
−1986	1994–1998	3.85E-11	43.686839
−1986	1998–2002	2.98E-50	222.213044
−1986	2002–	2.58E-95	429.079106
1986–1990	1990–1994	1.86E-03	9.686414
1986–1990	1994–1998	4.55E-29	125.224188
1986–1990	1998–2002	2.23E-105	475.318932
1986–1990	2002–	4.19E-175	795.907454
1990–1994	1994–1998	2.95E-28	121.510415
1990–1994	1998–2002	4.58E-135	611.783803
1990–1994	2002–	5.81E-230	1048.260856
1994–1998	1998–2002	4.53E-86	386.610058
1994–1998	2002–	7.34E-234	1066.196786
1998–2002	2002–	2.93E-101	456.39386

## 4 Results

### 4.1 Quantitative results

**Figure 1:**
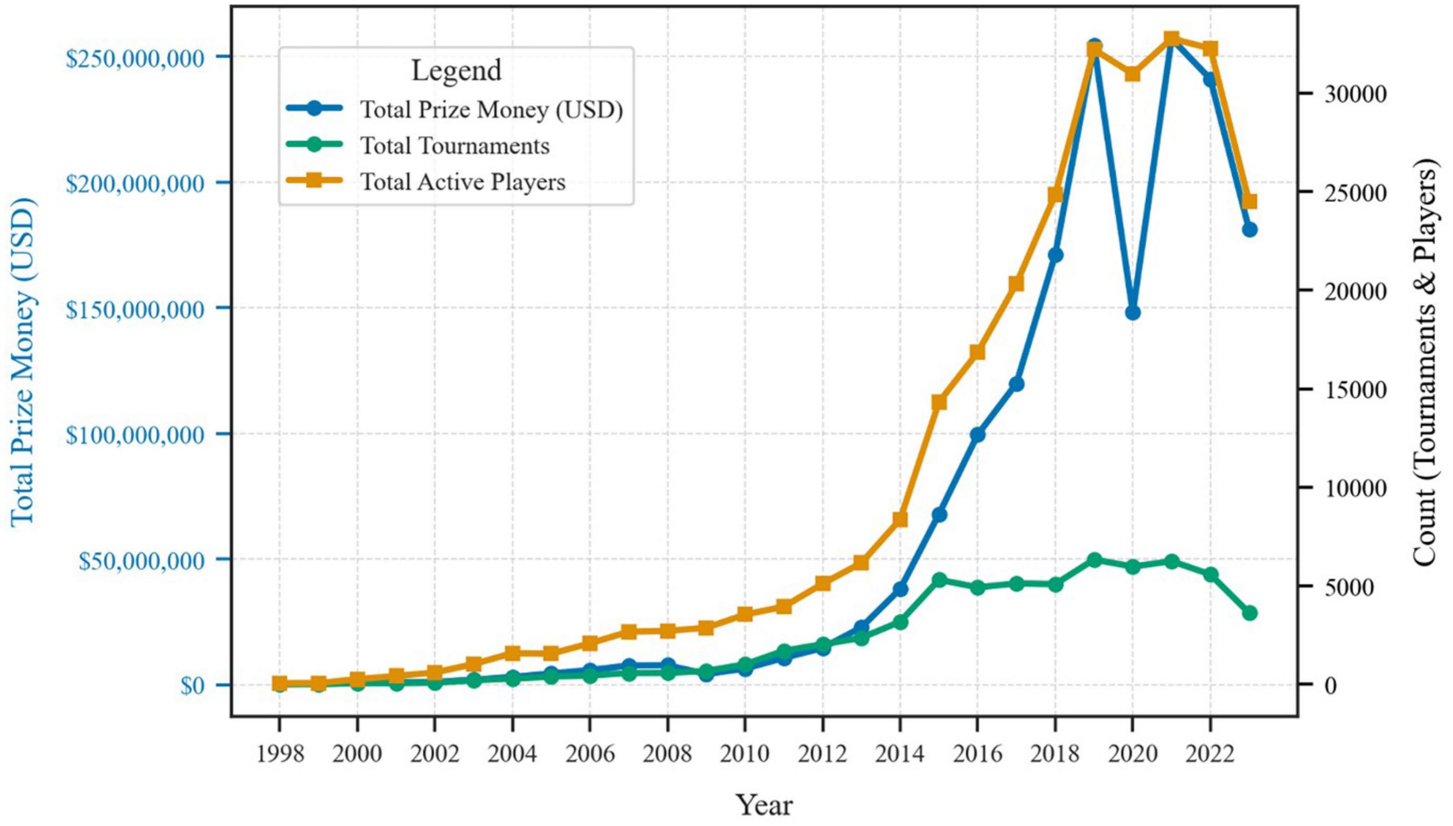
Annual trends in prize money, active players, and tournaments.

Figure 1 illustrates the exponential expansion of esports from 1998 to 2023, capturing annual totals for prize money, active competitors, and hosted tournaments. Initially, events were relatively few in number and offered modest prize pools; however, the industry soon witnessed a steep rise in investment and participation. By 2019, annual prize money exceeded USD 250 million, annual player base approached 30,000 active competitors, and more than 5,000 tournaments were hosted globally. The compound annual growth rate (CAGR) for prize money was 32.4% (95% CI [29.7, 35.1%]), significantly outpacing both player base growth (CAGR = 21.8, 95% CI [19.6, 24.0%]) and tournament growth (CAGR = 18.9, 95% CI [16.5, 21.3%]), all *p* < 0.001. Although these numbers dropped slightly in 2020, largely due to pandemic-related cancellations, they rebounded in subsequent years, underscoring the resilience and continued popularity of esports.

**Figure 2:**
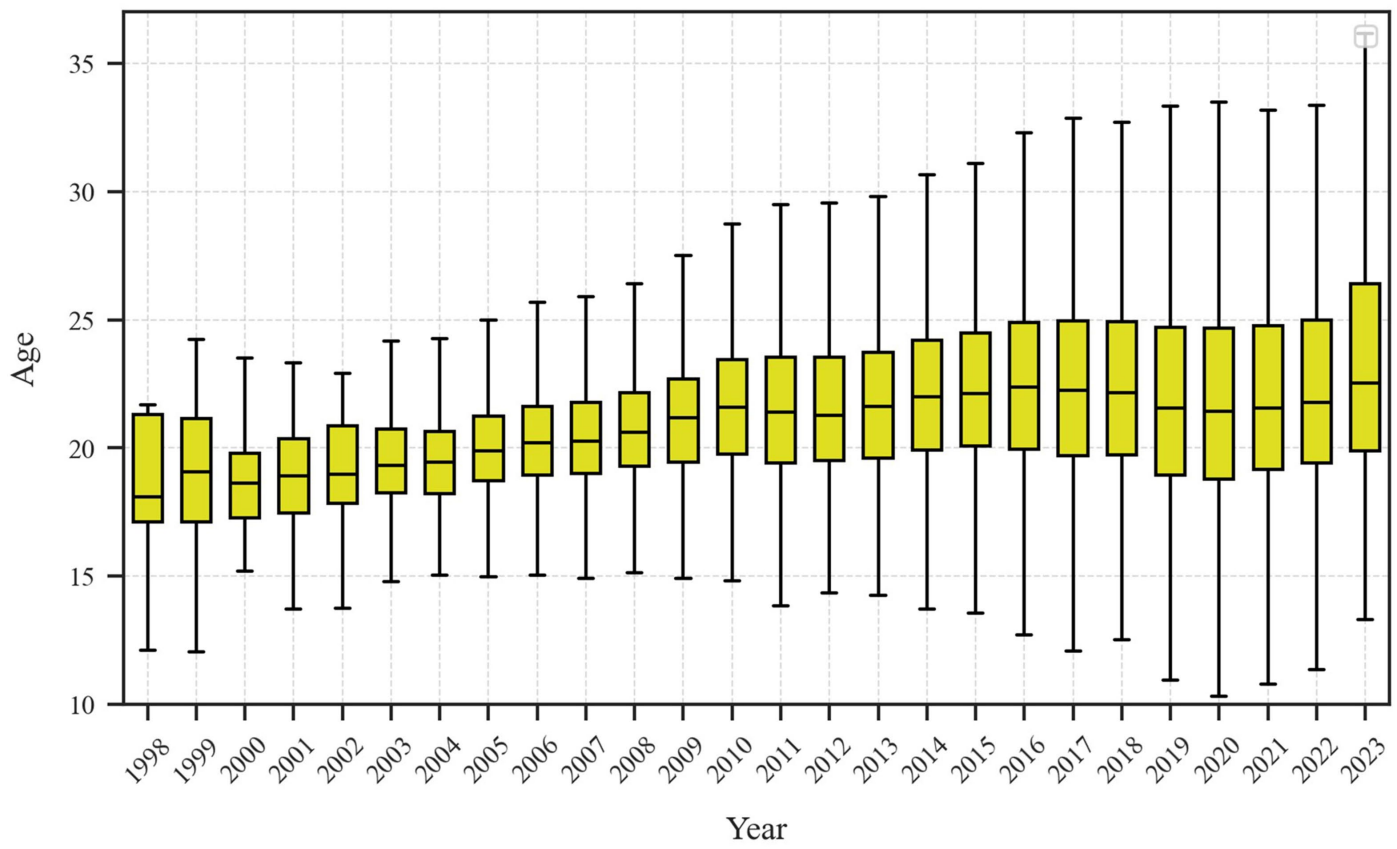
Age distribution of esports players over time.

While participation has broadened significantly, the player pool remains skewed toward younger age brackets. Figure 2 depicts the evolution of age distributions among esports competitors from 1998 to 2023, presented as annual box plots. In the early phase (1998–2006), the median age hovered around 18–19 years, with a relatively narrow interquartile range and a few outliers appearing in both younger and older brackets. From 2007 to 2015, the median age steadily climbed to about 21, and the upper age range began to extend toward the late 20s and early 30s. From 2016 onward, the median age stabilized around 22, but the dispersion in ages continued to widen, resulting in a more diverse player population. The widening of the quartile range suggests that while older players in their late 20s are staying active longer, youth continue to be the dominant demographic, with younger players participating more frequently than in the past.

**Figure 3:**
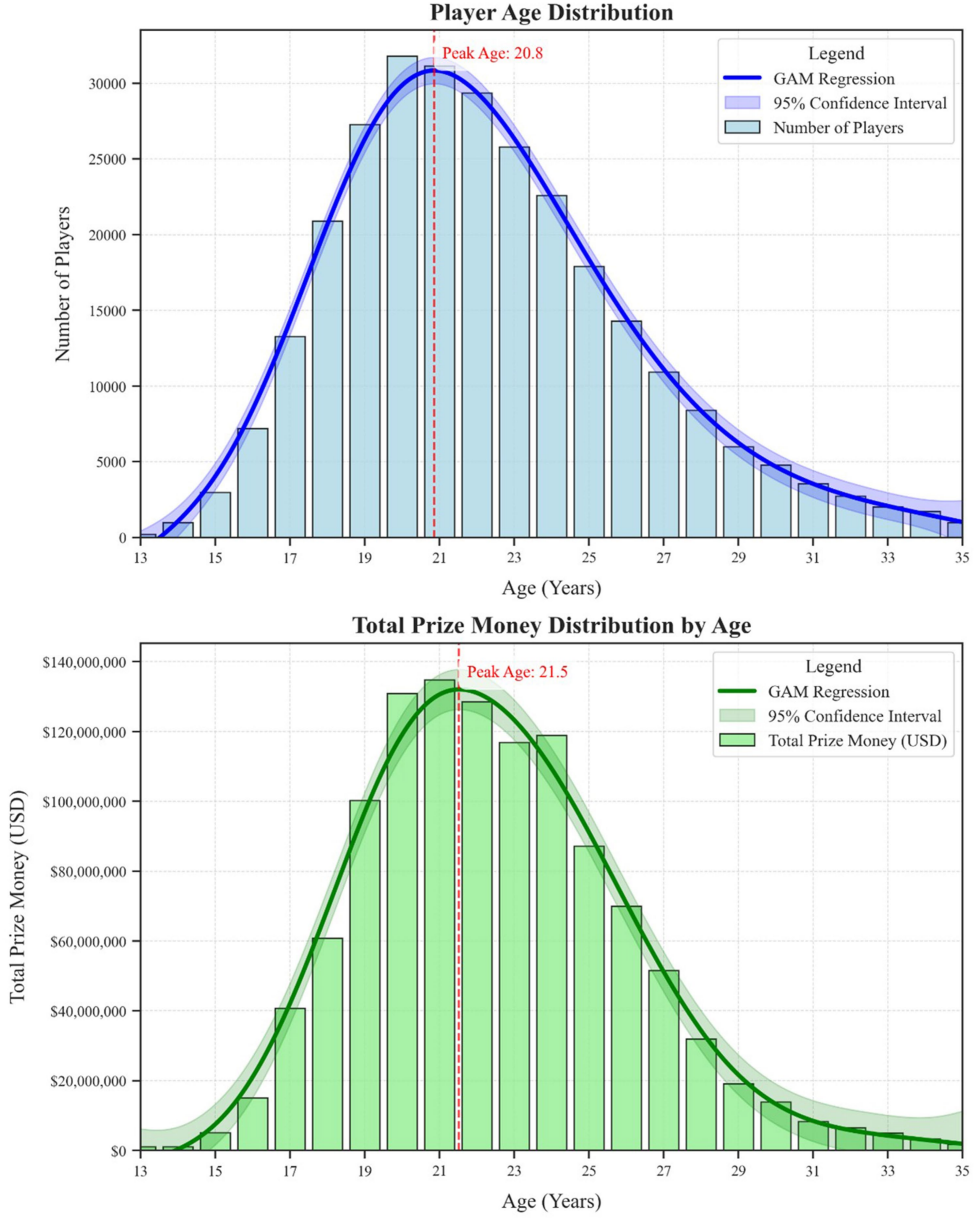
Player age distribution and prize money distribution by age.

Figure 3 compares the overall distribution of player ages with the total prize money earned across ages, revealing two slightly different peaks. The top panel, depicting the frequency of competitors, peaks at approximately 20.8 years of age, reflecting when most players are active. The bottom panel, depicting cumulative prize money by age, peaks around 21.5, indicating that while the largest number of competitors emerges around 20–21, maximum earning potential slightly lags behind the age at which players most frequently participate. This one-year difference could be attributed to the time needed to convert raw talent into high-level performance.

**Figure 4:**
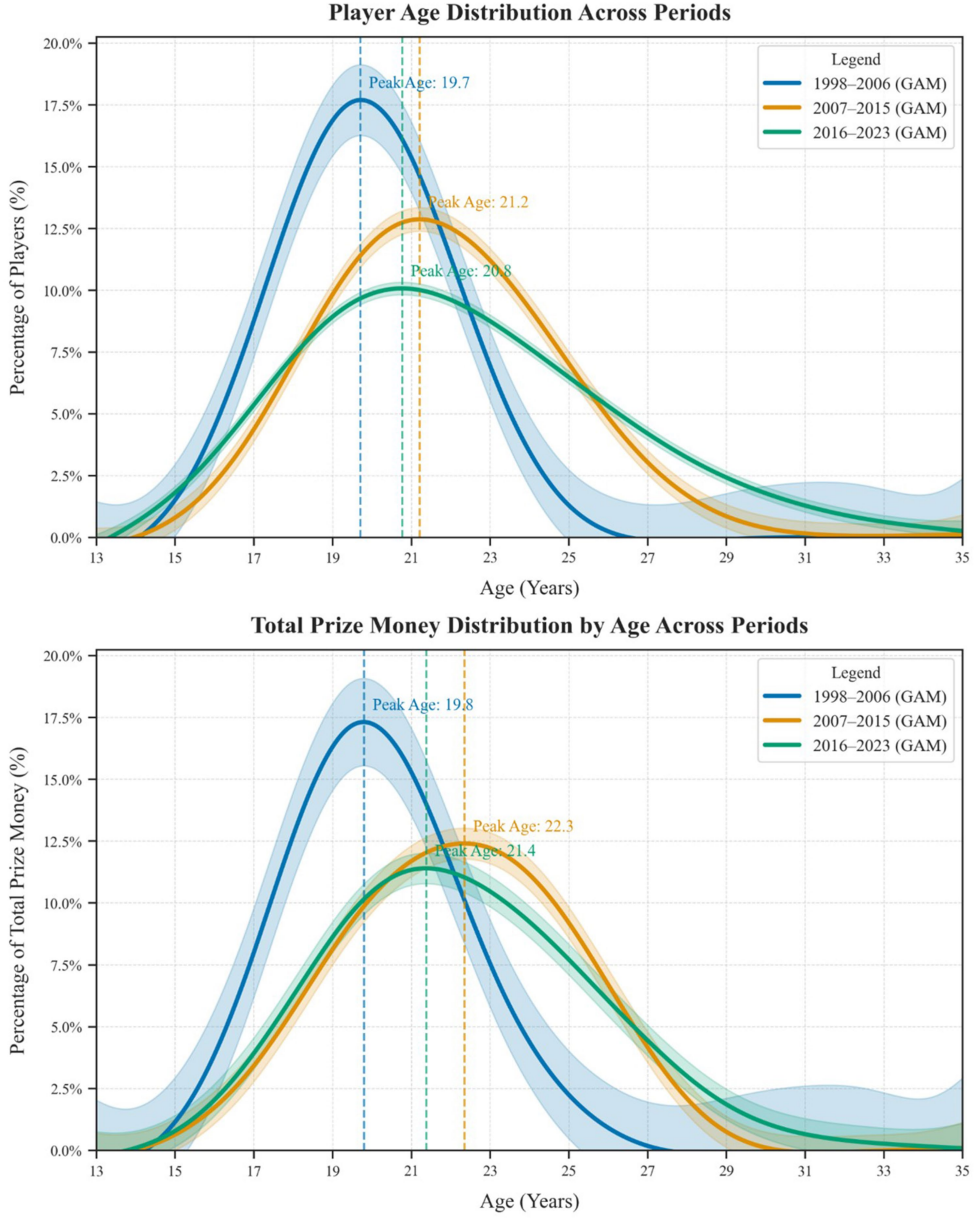
Comparative age and prize money distributions across three periods.

A more nuanced view emerges in Figure 4, which compares age and prize-money distributions across three distinct eras: 1998–2006, 2007–2015, and 2016–2023. These distributions reveal a progressive shift in competitive age windows. In the earliest period, the peak age for both participation and earnings was close to 19–20, accompanied by a more abrupt decline beyond the early 20s. Between 2007 and 2015, the peak moved to around 21–22, and more players were competing into their mid-20s. In the most recent period (2016–2023), the age distribution broadened further, with a peak near 21, an extended tail of older players, and a generally wider range of ages reaching notable prize-money thresholds. This temporal comparison implies that as the esports ecosystem matures through formalized training academies, better coaching, and greater financial backing, career opportunities are expanding for both up-and-coming teenagers and seasoned veterans. Nonetheless, the aggregate patterns still suggest a predominantly young demographic, with performance tending to taper off by the mid- to late-20s.

**Figure 5:**
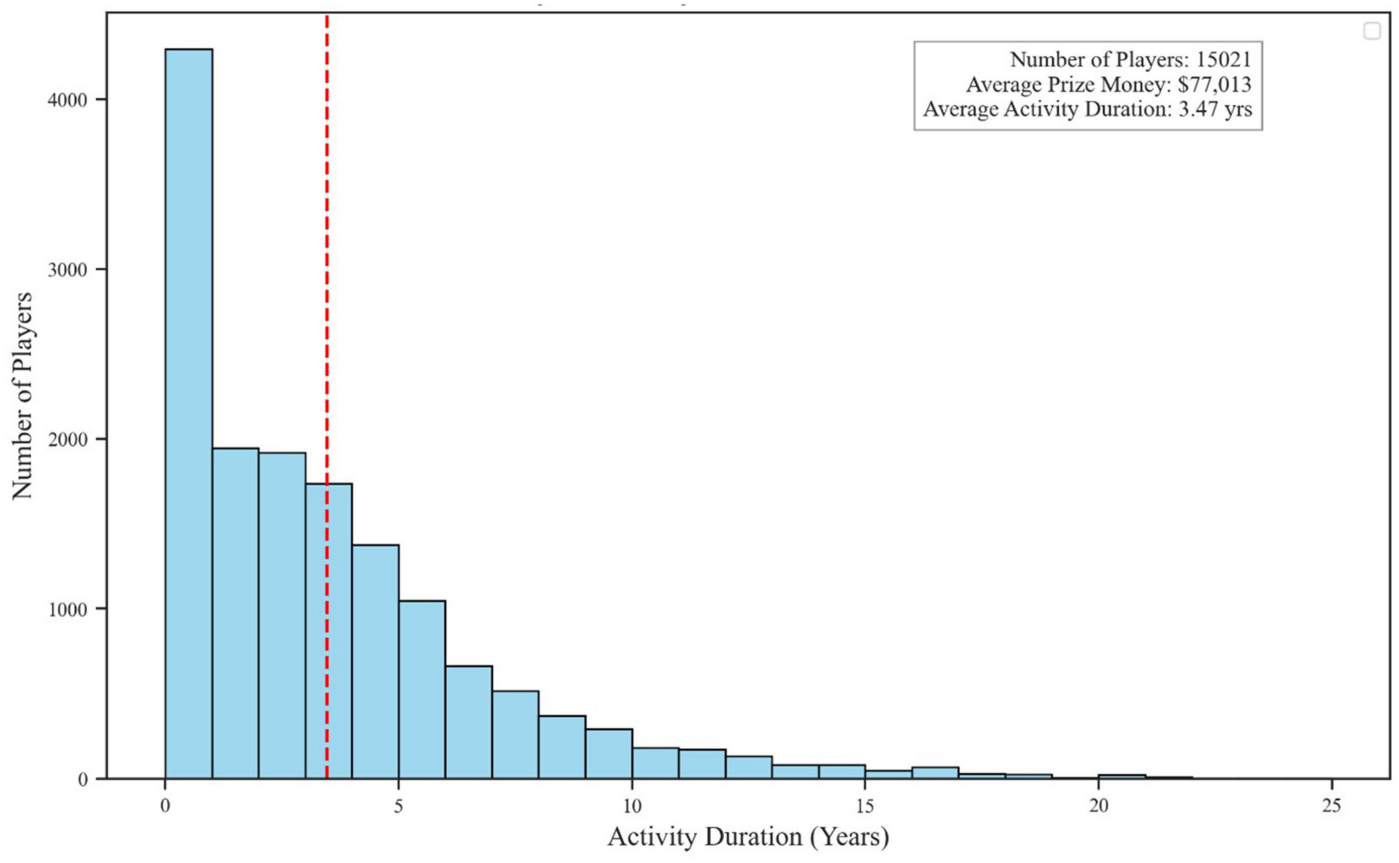
Distribution of career durations among esports players.

Figure 5 presents the overall distribution of career lengths. Although the average career spans 3.4 years, most players exit the professional scene within their first or second year. A minority maintain considerably longer tenures, some persisting for over a decade. These findings indicate a high-churn environment wherein short stints dominate, reflecting intense competitive pressure and uneven support structures for sustained participation.

**Figure 6:**
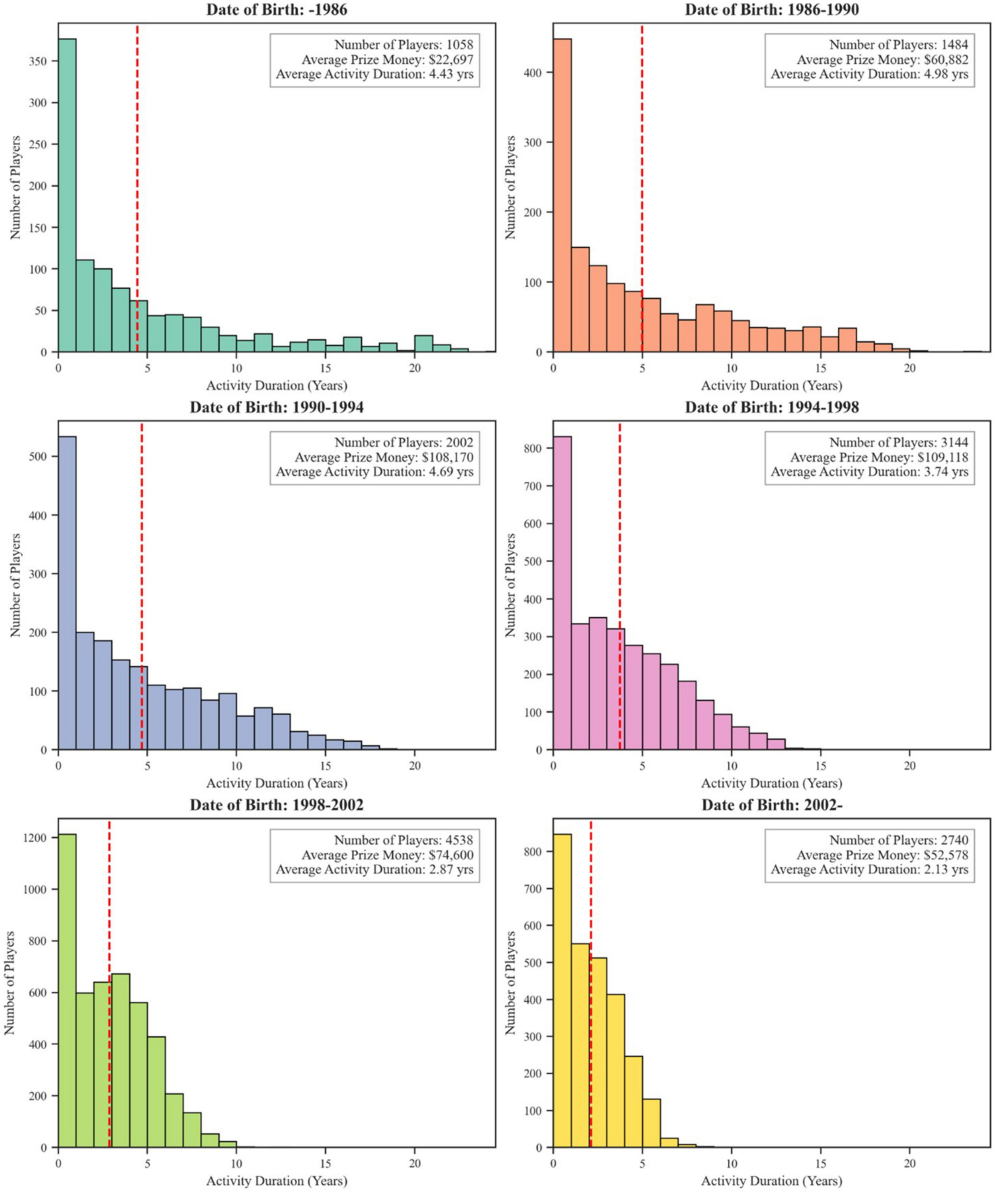
Career duration and earnings by birth cohort.

Figure 6 disaggregates career durations and total earnings by birth cohort. The three earliest cohorts (−1986, 1986–1990, and 1990–1994) exhibit remarkably similar patterns, with average activity durations of 4.43, 4.98, and 4.69 years, respectively. However, these cohorts differ markedly in earnings, with the average prize money increasing from $22,697 for the earliest group to $108,170 for the 1990–1994 cohort, reflecting the industry’s financial growth. A clear transition begins with the 1994–1998 cohort, where the average career duration decreases to 3.74 years. This shortening of careers is even more pronounced in the most recent cohorts: the 1998–2002 group averages just 2.87 years, while the post-2002 cohort shows the shortest average at 2.13 years. Notably, despite their briefer careers, the 1998–2002 cohort achieved the highest average prize money ($109,118), indicating a shift toward more concentrated periods of competitive success. This systematic shortening of career spans from the mid-1990s cohorts onward implies an increasingly compressed professional lifecycle in esports. This change suggests a transformation from the extended competitive careers seen in the first three cohorts to shorter, more intense periods of competition, although the post-2002 cohort’s shorter durations may partly reflect careers still in progress.

**Figure 7:**
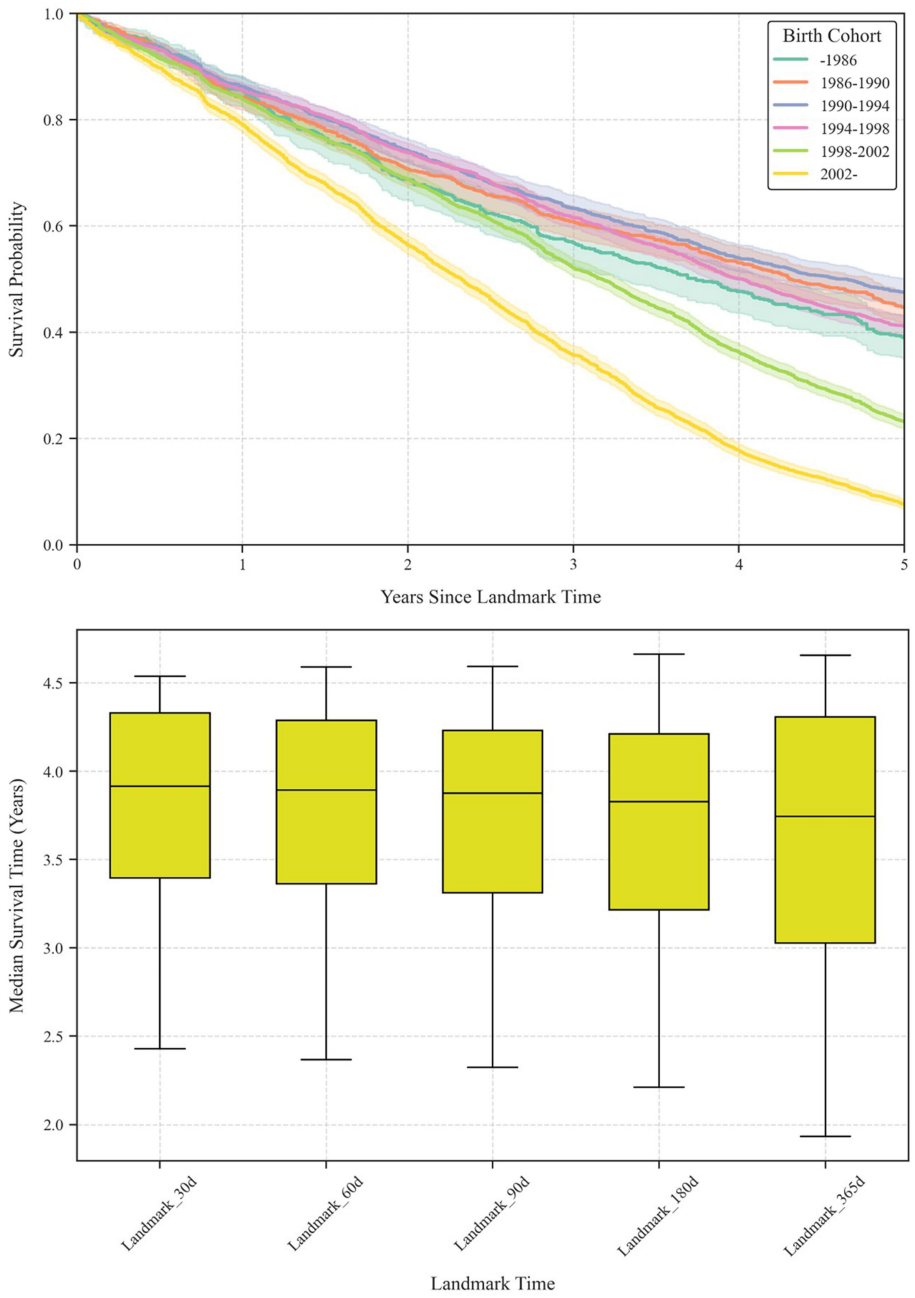
Kaplan–Meier survival analysis of esports career longevity.

Figure 7 further explores generational contrasts through Kaplan–Meier survival analysis. The upper panel displays 90-day conditional survival curves across the six birth cohorts, highlighting how participants from the four earliest cohorts (−1986, 1986–1990, 1990–1994, 1994–1998) demonstrate relatively higher retention rates over 5 years, whereas the two younger groups (1998–2002, 2002–) exhibit a steep drop-off: fewer than 20% of the latest cohort (2002–) remain active by the fourth year of their careers. These patterns persist under multiple landmark times (30, 60, 90, 180, 365 days), suggesting robust differences in career longevity (Table 3, Table 4).

### 4.2 Qualitative insights on dark aspects: burnout, exploitation, doping

Interviews with ten key stakeholders in the Korean esports ecosystem—professional and academy coaches, current and former players, organization managers, and an esports journalist—revealed a shared set of concerns regarding the more troubling sides of competitive gaming. While participants recognized the optimism fueled by rising prize pools and expanding recognition, they also highlighted systemic pitfalls in training, contracts, and doping enforcement that place additional strain on young talent (Holden et al., 2017).

#### 4.2.1 Burnout and excessive training

One recurring theme was burnout, particularly among teenage players. Many participants cited scrimmage schedules that stretch from morning until past midnight, with minimal breaks or supervised rest days. One academy coach recalled a 16-year-old recruit who “felt immense pressure to stay on the team, so he practiced until his wrists gave out” (Coach #2). Such anecdotes describe a culture in which early specialization, while instrumental for rapid skill acquisition, is accompanied by an intense training load that can degrade both mental and physical health (DiFrancisco-Donoghue et al., 2019). Several interviewees highlighted that without standardized player welfare guidelines or union representation, young competitors often perceive nonstop grinding as the only path to retaining their roster spot.

#### 4.2.2 Contractual exploitation and underage vulnerability

A second prominent issue involved exploitative contractual arrangements, especially at the academy and semi-professional levels. While larger, well-funded organizations have gradually begun to adopt legally vetted contracts, interviewees described a “wild west” dynamic outside elite circuits, where deals may be verbally brokered, contain unclear termination clauses, and provide little to no guaranteed salary. One former professional player summarized the problem: “If you are under 18, you do not know how to negotiate contracts, and the team knows it too” (Player #4). This imbalance of power entails heightened risk for young talent, especially given the prestige attached to signing with any recognizable esports organization. Acknowledging these issues, participants emphasized the need for industry-wide minimum standards, including transparent exit clauses and mandatory parental or legal oversight for minors.

#### 4.2.3 Doping and integrity loopholes

Although no interviewee admitted direct involvement in doping, many recognized stimulant misuse as a known practice in certain circles (Fashina, 2021). They traced this issue primarily to the decentralized nature of esports, where smaller tournaments often conduct few or no doping checks. One organization manager explained, “At big events, there’s some testing, but at smaller tournaments or online qualifiers, it’s basically the honor system” (Manager #6). This patchy enforcement creates opportunities for performance-enhancing substances to circulate unchecked; in addition, younger players striving to climb the professional ladder are especially susceptible to peer pressure and misinformation about “safe” stimulant use. Moreover, regarding integrity issues, participants described incidents of software-based cheating and match-fixing that sometimes go unpunished due to the lack of a unified sanctioning body (Scholz, 2019). While top-tier leagues may partner with external monitors like the Esports Integrity Commission, consistent application of integrity measures across all competitive tiers remains elusive.

Taken together, these qualitative findings align with the quantitative evidence of short, high-stress esports careers, illuminating how fragmented governance and an intense competitive ethos foster unhealthy conditions. Burnout emerges as a near inevitability for many teenage players, while inadequate contractual frameworks open the door to exploitation. Doping and cheating further threaten the credibility of esports—these issues mirror the integrity gaps seen in traditional sports but are magnified by the absence of a single, overarching regulatory authority (Holden et al., 2017). The picture that emerges is one of considerable promise coexisting with persistent structural failings—a reality that underscores the urgent need for reforms if esports is to evolve into a safer and more sustainable environment for its youngest talent.

### 4.3 Qualitative insights on light aspects: skill development, social capital, and opportunities

Although most of the interview narratives highlighted exploitative practices and the high risk of burnout, several participants also described positive, even transformative, experiences within the esports ecosystem. These “light” aspects primarily concerned skill cultivation, supportive team structures, and the sense of community that can emerge among players who share a passion for competitive gaming (Zhong et al., 2022).

#### 4.3.1 Skill development and transferable expertise

Several interviewees emphasized that rigorous, deliberate practice in esports can cultivate valuable cognitive and interpersonal capabilities—benefits that often remain underappreciated outside professional circles. One analyst remarked, “Our players’ reflexes and strategic thinking are on another level, but that mindset carries over to teamwork and problem-solving outside the game” (Analyst #5). Coaches similarly noted that certain training drills not only enhance reaction speeds but also promote disciplined goal-setting and collaboration. These insights challenge the stereotype that gamers are merely “wasting time” and demonstrate that structured practice in a supportive environment can yield both successful in-game performance and broader life skills.

#### 4.3.2 Organizational support and mentorship

While inconsistent governance remains a pervasive issue, a handful of well-resourced teams have begun to adopt more holistic athlete support programs. One academy coach cited weekly wellness sessions, mandatory rest days, and mental health counseling as best practices introduced by sponsors seeking to protect their investments in top prospects (Coach #3). Another participant, a former professional coach, explained how mentorship programs connect experienced veterans with younger recruits: “We do not just focus on the game; we talk about balancing school, social life, and managing online toxicity. It’s not perfect, but it’s a start” (Coach #10). These examples illustrate that certain organizations view sustainable player development as integral to long-term success, mitigating at least some of the structural pressures that lead to rapid burnout.

#### 4.3.3 Community and personal fulfillment

Several interviewees recalled moments of genuine camaraderie and personal achievement that overshadowed the challenges of early specialization. As one active player in a secondary league described, “There’s a tight-knit feeling here. Even though the pay is not huge, we share strategies, celebrate each other’s wins, and try to stay positive” (Player #8). Such expressions of mutual support align with broader findings in media psychology that online gaming communities can foster resilience and social capital (Granic et al., 2014; Macey and Hamari, 2019). Although these experiences did not dominate this study’s interviews—likely because participants were more inclined to discuss systemic problems—they highlight the potential for esports to offer not just competitive excitement but also meaningful social connections and personal growth.

Overall, although the qualitative data largely spotlighted the structural challenges that contribute to high turnover and early burnout, it also revealed notable pockets of constructive practice. Supportive team policies, opportunities for skill transfer, and strong community ties can help offset the “dark” pressures of relentless competition and fragmented governance. The duality of these experiences reinforces that esports is neither inherently detrimental nor purely beneficial; its impact on young athletes depends significantly on whether robust ethical standards, balanced training regimens, and comprehensive support structures are in place.

## 5 Discussion

### 5.1 Overview of key findings

The quantitative and qualitative strands of this study converge to portray an esports environment that is both teeming with opportunities and fraught with high risks for its youngest participants. On the one hand, increasing prize pools and professionalization signal a “light” side, encompassing rapid skill acquisition, social capital, and potentially lucrative career paths (Zhong et al., 2022; Ahn et al., 2020). On the other hand, a “dark” side surfaces in the form of fragmented governance, strenuous training regimens, and exploitative contracts, elements that collectively contribute to short and volatile careers (Kari and Karhulahti, 2016; DiFrancisco-Donoghue et al., 2019; Poulus et al., 2024a; Cote et al., 2023).

From the quantitative perspective, while newer cohorts are evidently experiencing more intense competition and higher financial gains, they are also exiting professional esports earlier than their predecessors. This trend suggests that while the potential rewards have grown, the structural safeguards needed to sustain a longer career—such as standardized rest periods, mental health support, and fair contract enforcement—have not kept pace (Holden et al., 2017). In essence, the industry’s rapid expansion appears to magnify both the “light” and “dark” dimensions of early specialization: players are reaching higher peaks faster but also encountering burnout and career instability more frequently.

Our qualitative interviews reinforce these patterns. Interviewed stakeholders described a landscape in which scrimmages can exceed 10 h per day, while legal protections for underage competitors are often minimal or inconsistently enforced (Bulut, 2020). Despite these concerns, pockets of constructive practice have emerged. Certain well-funded organizations and supportive team cultures point to an alternative path—one where regular breaks, mentorship programs, and comprehensive health services can begin to offset the mental and physical toll. This dichotomy illustrates that the dividing line between a positive or negative esports experience is not inherent to the medium itself but rather mediated by governance structures and team-level approaches to player welfare (Salum et al., 2024; Scholz, 2019; Legault and Weststar, 2017).

Overall, these dual findings suggest an industry at a crossroads. The escalating popularity and commercial clout of esports underscore its potential to be a global force for digital innovation and social engagement—the “light” side. At the same time, limited oversight and fragmented regulatory systems threaten to undermine these gains by perpetuating overtraining, doping, and contract exploitation—the “dark” side (Fashina, 2021; Macey and Hamari, 2019). Consequently, a critical juncture has been reached: esports must reconcile its promise of rapid skill development and community building with the practical realities of sustaining athlete well-being if it is to evolve into a truly sustainable, reputable sector of competitive entertainment.

### 5.2 Policy and governance recommendations

This study’s findings underscore the urgent need for comprehensive reforms in esports governance that can reconcile the industry’s rapid growth with the welfare of its youngest participants. The pervasive issue of inconsistent and often exploitative contractual arrangements has left many young players vulnerable, highlighting the necessity of industry-wide standards. A unified approach to contract formulation could mandate clear termination clauses, establish baseline pay or stipend requirements, and ensure that parental or legal oversight is integral when minors are involved. Such measures could not only protect players from arbitrary dismissal but also promote fairer, more transparent working conditions.

In parallel, addressing the high rates of burnout observed among players requires a rethinking of training practices and support mechanisms. Drawing on best practices from traditional sports medicine, esports organizations should integrate mandatory rest days and structured wellness programs into their operational models. For example, access to on-site physiotherapy, mental health counseling, and nutritional guidance could help mitigate the intense physical and psychological pressures that characterize high-level competition. By investing in holistic player development, the industry can help extend career longevity and improve overall well-being, thereby transforming some of the inherent risks of early specialization into opportunities for sustainable growth.

Furthermore, the fragmented nature of the current anti-doping and integrity measures highlights the need for a centralized regulatory framework. While top-tier events may collaborate with bodies such as the Esports Integrity Commission, many smaller tournaments remain largely unregulated, which fosters an environment where unethical practices can proliferate. A cohesive regulatory body, working collaboratively with game publishers and existing oversight organizations, could ensure consistent testing procedures and enforceable sanctions across all levels of competition. This unified approach could not only safeguard fair play but also enhance the credibility and ethical standing of the entire esports ecosystem.

Finally, fostering ongoing dialogue among all stakeholders is essential. To this end, regular stakeholder forums involving players, coaches, team owners, and government agencies could provide a platform for sharing best practices and updating policies based on emerging challenges and successes. Such collaborative efforts could facilitate the continual refinement of governance standards, ensuring that the positive potentiality of esports—for example, its capacity to develop skills, foster community, and drive innovation—is fully realized while its darker aspects are effectively mitigated.

### 5.3 Implications for psychology and media research

The dual “light” and “dark” dimensions of esports reflect broader debates in media psychology about the consequences of intensive digital engagement. Scholars have long recognized both the potential for skill acquisition and social enrichment in gaming (Granic et al., 2014) as well as the attendant risks of addiction, anxiety, and other adverse outcomes (Kowert, 2020). Our findings expand this dialogue by underscoring how structural factors—namely, fragmented governance, performance pressures, and underdeveloped athlete support systems—can amplify or attenuate these psychological effects in high-stakes competitive environments.

First, the evidence of shortened professional trajectories underscores the importance of longitudinal approaches to digital gaming research. Rather than focusing on isolated effects or short-term measures of engagement, future studies could adopt extended observation windows or panel designs to track how psychological well-being, social bonds, and cognitive outcomes evolve over time (Macey and Hamari, 2019). Such designs could illuminate whether players who receive structured physical and mental health support are more likely to experience sustainable skill development and lower burnout rates.

Second, this study’s qualitative insights into both exploitative and supportive organizational practices highlight the role of social contexts in shaping gaming experiences. While early specialization can provide a sense of purpose and identity, it may also precipitate social isolation and vulnerability if players lack basic protections and communal resources (DiFrancisco-Donoghue et al., 2019). Conversely, well-resourced teams that incorporate mentorship programs and mental health coaching exemplify how social capital and positive group norms can moderate stressors. Recent research confirms this dynamic, demonstrating that players with robust social support networks and adaptive coping strategies show greater resilience against burnout in high-level competition (Poulus and Polman, 2022; Leis et al., 2024).

Finally, these findings contribute to ongoing discussions about ethical design and governance in digital platforms. In the case of esports, if game publishers and esports leagues do not address doping, contract ambiguities, and inconsistent welfare support, the psychological costs for players—particularly minors—could overshadow the documented cognitive and social benefits of gaming (Kowert, 2020; Salum et al., 2024). Esports-adapted psychological training significantly enhances resilience and mental health among elite players (Poulus et al., 2023). Researchers should, therefore, examine the impact of policy interventions such as mandatory rest days or robust anti-doping enforcement on player attitudes, motivation, and mental health outcomes. Moving forward, interdisciplinary collaborations among media psychologists, policymakers, and industry stakeholders will be crucial for translating scholarly insights into tangible reforms, ensuring that esports can fully realize its potential as a dynamic and socially beneficial digital medium.

### 5.4 Limitations and future research

Despite this study’s valuable insights, several limitations must be acknowledged that can pave the way for future research. One primary concern relates to the data sources and the representativeness of our quantitative dataset. By relying on public aggregator websites such as *Esports Earnings* and *Liquipedia*, our analysis predominantly reflects high-profile tournaments and well-documented players. Consequently, smaller regional competitions and lower-tier players may be underrepresented, potentially biasing our findings toward more prominent figures in the esports landscape.

Furthermore, the historical nature of the dataset, spanning 25 years, introduces inherent challenges related to data quality and the consistency of record-keeping. For example, the operational definition of “career duration”—measured according to documented tournament participation—might misclassify temporary breaks, such as those due to education or mandatory service, as career terminations. Such misclassifications may lead to an underestimation of true career longevity and obscure the nuances of intermittent career trajectories.

The qualitative component of our study, while rich in detail, also presents limitations. Our focus on stakeholders within the Korean esports ecosystem provides critical insights into a leading market; however, Korea’s unique regulatory, cultural, and market dynamics may not fully capture the global diversity of esports practices. Regional differences in regulatory frameworks create varied player experiences (Harris et al., 2022; Wilson et al., 2024; Cote et al., 2023). The Korean approach to esports governance, with its greater government involvement compared to Western markets, may produce findings that do not directly translate to other contexts. Additionally, our relatively small sample size of 10 participants, though purposively selected to represent a range of roles, limits the generalizability of these findings. Broader participation, particularly from league administrators, policymakers, and sports medicine professionals, would likely yield a more comprehensive understanding of the multifaceted challenges within the industry.

Our measurement methodology introduces additional constraints. Defining “career duration” solely through tournament participation may misrepresent careers with temporary hiatuses for education, military service (particularly relevant in Korea where service is mandatory for males), or health-related breaks. Similarly, our focus on prize money excludes crucial income streams like salaries, sponsorships, and streaming revenue that increasingly define financial success in contemporary esports.

Looking ahead, future research should incorporate a wider array of data sources, including those capturing regional and lower-tier tournaments, to offer a more representative picture of the esports ecosystem. Longitudinal and panel studies that track players over extended periods, including instances of temporary breaks, could provide a deeper understanding of career trajectories and transitions, such as shifts from competitive play to roles in coaching or broadcasting. Comparative studies across different geographic regions and cultural contexts could also illuminate how varying governance structures and societal norms influence career longevity. In addition, integrating physiological and performance metrics with existing data could enrich our understanding of the physical and mental tolls associated with early specialization. Finally, intervention-based research evaluating the effectiveness of standardized contractual frameworks, holistic athlete support systems, and centralized anti-doping measures could offer critical insights into the potential for policy reforms to mitigate the risks identified in our study.

Building on promising recent work, researchers should expand investigations into physical training’s impact on career sustainability. Some have begun exploring relationships between structured exercise, cognitive function, and performance in esports—a direction that may yield valuable insights into reducing injury rates and enhancing cognitive sustainability (McNulty et al., 2023; Nicholson et al., 2024a; Nicholson et al., 2024b). Similarly, controlled trials on psychological interventions could establish evidence-based protocols for mental health support in professional esports settings (Poulus et al., 2023).

By addressing these limitations and exploring these future research directions, subsequent investigations can build a more nuanced and comprehensive understanding of the factors influencing career longevity in esports. Such insights will be instrumental in guiding industry reforms and developing targeted interventions that enhance both the sustainability of esports careers and the well-being of its athletes.

## 6 Conclusion

This study illuminates the dual nature of early specialization in esports by integrating quantitative analyses of career trajectories and earnings with qualitative insights from key stakeholders in the Korean esports ecosystem. Our findings reveal that while the rapid professionalization of esports offers significant opportunities—such as accelerated skill development, heightened social capital, and rapid financial gains—it is concurrently marred by high rates of burnout, exploitative contractual practices, and inconsistent regulatory oversight. The stark contrast between the “light” and “dark” sides of gaming underscores that the very practices that propel young players to early success also predispose them to premature career termination and adverse health outcomes (Scholz, 2019; DiFrancisco-Donoghue et al., 2019; Poulus et al., 2024a).

The quantitative data demonstrate that while prize pools and participation levels have soared over recent decades, newer cohorts have significantly shorter professional careers compared to their older counterparts. Qualitative accounts further illuminate this paradox, highlighting both the benefits of rigorous, skill-enhancing training and the deleterious effects of relentless practice schedules, insufficient welfare provisions, and fragmented governance. These insights collectively indicate an urgent need for systematic reforms—including standardized contractual safeguards, comprehensive player welfare programs, and centralized integrity oversight—to nurture a more sustainable and ethically sound esports environment.

In contributing to the broader discourse in media psychology and digital gaming research, this study emphasizes that esports is neither inherently detrimental nor beneficial. Instead, its impact on young athletes largely depends on the surrounding organizational and regulatory frameworks. Future research can extend these findings through longitudinal and cross-cultural approaches that examine the complex interplay between technological innovation, governance practices, and player well-being. Particular attention to the unique power dynamics where game publishers control competitive environments—unlike traditional sports—will be essential for understanding how these dynamics shape career development and sustainability.

Ultimately, our findings advocate a balanced approach that maximizes the “light”-side benefits of esports, such as cognitive and social development, while mitigating the “dark”-side risks of exploitation and burnout. Addressing these challenges is crucial not only for safeguarding the futures of individual players but also for ensuring the long-term credibility and sustainability of esports as a global competitive phenomenon.

## Data Availability

The raw data supporting the conclusions of this article will be made available by the authors, without undue reservation.
