# Supplementary material for: Game over too soon: early specialization and short careers in esports
Source: Front Psychol. 2025 May 12;16:1585599. doi: 10.3389/fpsyg.2025.1585599 (PMC12104221; doi:10.3389/fpsyg.2025.1585599)
Supplement: Supplementary file 1 [file Data_Sheet_1.PDF]

Supplementary Material

1 Data Cleaning Flowchart

Below is a textual representation of the data cleaning flowchart used to transform the initial raw dataset into the final analytic sample of 15,021 unique esports player profiles. This flowchart outlines the key steps and decision points in our data processing pipeline.

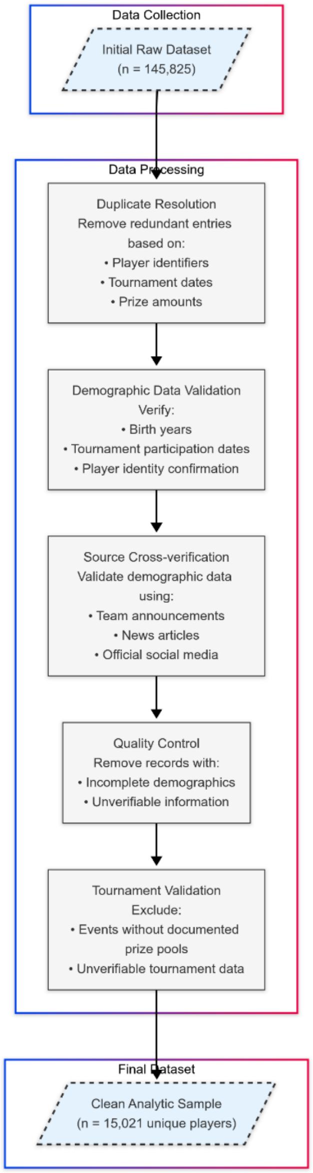

Supplementary Figure 1. Data Cleaning Flowchart

2 Interview Protocol

The semi-structured interview protocol was developed to explore key themes related to governance, early specialization, burnout, and integrity challenges in the Korean esports ecosystem. The protocol was designed to be flexible, allowing interviewers to probe for additional details and clarifications as needed while ensuring that all core topics were addressed. Below is an outline of the protocol:

1. **Introduction**
   - **Purpose:** Explain the purpose of the study, emphasizing the exploration of career trajectories, governance practices, and the challenges faced by esports stakeholders.
   - **Confidentiality:** Assure participants that their identities will remain confidential and that all data will be anonymized.
   - **Format:** Describe the interview format (e.g., face-to-face or via secure video conferencing) and approximate duration (15–60 minutes).
2. **Background Information**
   - **Role and Experience:** Ask participants to briefly describe their role within the esports ecosystem, including their professional background and current responsibilities.
   - **Context Setting:** Request a brief overview of their experience with early specialization and how they perceive its impact on player development.
3. **Core Themes and Questions**
   - **Governance and Contractual Practices**
     - *Sample Questions:*
       - "Can you describe the nature of the contracts that players typically sign in your organization?"
       - "How do you view the current governance structures in esports? What challenges do you face with regulatory oversight?"

        - "In your experience, how effective are current efforts (e.g., by KeSPA or other bodies) in enforcing fair contractual practices?"
- **Early Specialization and Burnout**
    - *Sample Questions:*
        - "What does early specialization mean in the context of your experience in esports?"
        - "Can you share an example of how intensive training or early specialization has impacted a player's career?"
        - "What measures, if any, does your organization take to address the risks of burnout and physical or psychological strain?"
- **Doping and Integrity**
    - *Sample Questions:*
        - "Have you observed any issues related to performance-enhancing substances or integrity challenges in your field? If so, can you elaborate?"
        - "How consistent are anti-doping measures across different levels of competition within the industry?"
        - "What do you believe could be done to improve the integrity of competitive play in esports?"
- **General Reflections and Future Directions**
    - *Sample Questions:*
        - "Based on your experience, what reforms would you recommend to improve the overall sustainability of esports careers?"

        - "How do you envision the future of governance and player support in esports evolving in the coming years?"

4. **Closing**
    - **Final Comments:** Invite participants to share any additional thoughts or experiences that were not covered during the interview.
    - **Next Steps:** Explain how the data will be used and offer to share a summary of the findings with participants if they are interested.
    - **Thank You:** Express appreciation for the participant's time and insights.
